# Supplementary material for: VGEA: an RNA viral assembly toolkit
Source: PeerJ. 2021 Sep 6;9:e12129. doi: 10.7717/peerj.12129 (PMC8428259; doi:10.7717/peerj.12129)
Supplement: Supplemental Information 2 [file peerj-09-12129-s002.zip › Supplementary_File_2/Performance_Comparison_Assembly_Pipelines_Table_2/CV45/vgea/quast_results/results_2021_05_13_23_04_04/icarus_viewers/alignment_viewer.html]

|  |  |  |
| --- | --- | --- |
| Main menu  Icarus  **QUAST Contig Browser** | Move << < > >> zoom +5x +2x –2x –5x \| start end Show misassemblies: relocations (0) translocations (0) inversion (1) local (0) | Search contig or gene: |

**Contig alignment viewer**. Contigs aligned to NC 045512.2

Hide
Show annotation
Hide
Show read coverage and GC distribution
Show physical
Show physical
Hide GC %
Hide GC %

+
-
Reset

+
-
Reset
